# Software for the high-throughput collection of SAXS data using an enhanced *Blu-Ice*/*DCS* control system

**DOI:** 10.1107/S0909049510028566

**Published:** 2010-09-03

**Authors:** Scott Classen, Ivan Rodic, James Holton, Greg L. Hura, Michal Hammel, John A. Tainer

**Affiliations:** a Physical Biosciences Division, Lawrence Berkeley National Laboratory, Berkeley, CA 94720, USA; b Life Sciences Division, Lawrence Berkeley National Laboratory, Berkeley, CA 94720, USA; c Department of Biochemistry and Biophysics, University of California, San Francisco, CA 94158-2330, USA

**Keywords:** SAXS, software, beamline, control system, *Blu-Ice*, *DCS*, SIBYLS, GUI

## Abstract

Biological small-angle X-ray scattering (SAXS) provides powerful complementary data for macromolecular crystallography (MX) by defining shape, conformation and assembly in solution. Although SAXS is in principle the highest throughput technique for structural biology, data collection is limited in practice by current data collection software. Here the adaption of beamline control software, historically developed for MX beamlines, for the efficient operation and high-throughput data collection at synchrotron SAXS beamlines is reported. The *Blu-Ice* GUI and *Distributed Control System* (*DCS*) developed in the Macromolecular Crystallography Group at the Stanford Synchrotron Radiation Laboratory has been optimized, extended and enhanced to suit the specific needs of the biological SAXS endstation at the SIBYLS beamline at the Advanced Light Source. The customizations reported here provide a potential route for other SAXS beamlines in need of robust and efficient beamline control software. As a great deal of effort and optimization has gone into crystallographic software, the adaption and extension of crystallographic software may prove to be a general strategy to provide advanced SAXS software for the synchrotron community. In this way effort can be put into optimizing features for SAXS rather than reproducing those that have already been successfully implemented for the crystallographic community.

## Abstract 2

The *Blu-Ice* GUI and *Distributed Control System* (*DCS*) developed in the Macromolecular Crystallography Group at the Stanford Synchrotron Radiation Laboratory has been optimized, extended and enhanced to suit the specific needs of the SAXS endstation at the SIBYLS beamline at the Advanced Light Source. The customizations reported here provide one potential route for other SAXS beamlines in need of robust and efficient beamline control software.

## 1. Introduction

Although it was demonstrated as early as 1935 that the hydrodynamic radius of proteins could be determined by light scattering (Putzeys & Brosteaux, 1935 ▶), the adoption of SAXS (small-angle X-ray scattering) as a technique for exploring complex biological systems has exploded in the past 10–20 years. Besides the SIBYLS beamline (Trame *et al.*, 2004 ▶) at the Advanced Light Source there are numerous synchrotron beamlines now in operation that specialize in collecting data from biological samples, including, but not limited to, BioCAT at the APS, BL4.2 at SSRL, beamline X33 at EMBL/DESY, beamlines ID2, ID14-3 and BM26 at ESRF, SWING at SOLEIL, beamline 5.2L at ELETTRA, and beamline I22 at Diamond. A further indication of the growing acceptance of SAXS by the broader biosciences community is the number of papers in the PubMed database that contain ‘SAXS’ in the title/abstract, which has gone from 14 in 1997 to 205 in 2009, over a ten-fold increase in 12 years. These numbers do not include papers that incorporate SAXS data without including it in the titles and abstracts so the actual number of ‘SAXS’ papers is likely to be much greater. The emergence of biological SAXS has necessitated the development of more accessible data collection software. Importantly, many of the most powerful applications of SAXS involve its combination with X-ray crystallography (Putnam *et al.*, 2007 ▶) including the characterization of flexible structures (Tsutakawa *et al.*, 2007 ▶; Pelikan *et al.*, 2009 ▶) suggesting that optimized SAXS data collection software based on the well known *Blu-Ice*/*DCS* system would be readily adoptable by crystallographers as well as other biological SAXS researchers. Indeed, with advanced synchrotron facilities, SAXS has proven especially powerful to complement crystallography by providing information on dynamic assemblies and conformational changes, as shown by recent SAXS results on the DNA-dependent protein kinase complex acting in joining DNA ends (Hammel *et al.*, 2010 ▶) and on RNA structures (Rambo & Tainer, 2010*b* ▶). Consequently current structural biology reviews highlight the importance of SAXS combined with other methods for accurate solution structures and structures of dynamic biological complexes (Rambo & Tainer, 2010*a* ▶; Perry *et al.*, 2010 ▶).

At the SIBYLS beamline recent advances in the automation of SAXS data collection (Hura *et al.*, 2009 ▶) have driven the development of software tools to collect high-throughput data. The SIBYLS beamline is a dual-endstation beamline having both world-class SAXS and macromolecular crystallography (MX) endstations. The initial design of the SIBYLS beamline envisioned highly cooperative interactions between MX and SAXS both at the scientific and technological levels. It was hoped that SAXS, being an emerging technique, would benefit greatly from sharing a beamline with the more well established technique of crystallography. In the spirit of this cooperative vision we have undertaken the task of leveraging a beamline control system from MX for use with the SAXS endstation. The SIBYLS MX endstation has adopted the *Blu-Ice*/*DCS* beamline control software originally developed at the SSRL (McPhillips *et al.*, 2002 ▶; Soltis *et al.*, 2008 ▶). Recently we have installed a Hamilton MICROLAB 4000 liquid-handling robot that is capable of transferring SAXS samples (10–20 µl) from a 96-well plate to the sample cell. Additionally, the Hamilton robot has been programmed to discard used samples and wash/rinse the sample cell between uses. Until recently the Hamilton robot was controlled *via* a separate computer using vendor-provided software. We have now extended and enhanced the functionality of the *Blu-Ice*/*DCS* system to enable the collection of high-throughput and automated SAXS data, thus interleaving robot control with sample X-ray exposure and detector control. Similar efforts have been undertaken at other synchrotron SAXS beamlines; for example, at the X33 station at EMBL (Hamburg, Germany) (Round *et al.*, 2008 ▶), the SWING beamline at SOLEIL (Paris, France) (David & Pérez, 2009 ▶), beamline 4-2 at SSRL (Stanford, USA) and the ID14-3 bio-SAXS beamline at ESRF (Grenoble, France). At first glance this might appear to be a local solution to a local problem. However, we believe that by presenting our solution we will share with the broader synchrotron community alternate strategies and methods to approach system integration tasks.

**Figure 1:**
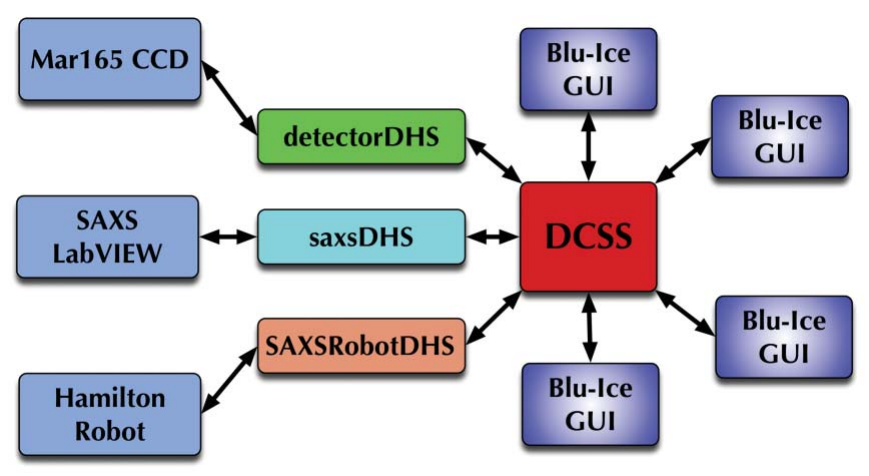
Schematic diagram illustrating the organization of the *DCS* Server (DCSS), the Distributed Hardware Servers (DHSs) and the *Blu-Ice* GUI connections.

The *Distributed Control System* (*DCS*) is a beamline control system capable of coordinating hardware and software communications in a large and heterogeneous environment. The *DCS* server (DCSS) is the central control hub that communicates with the peripheral control computers *via* distributed hardware servers (DHSs). A DHS is specialized software that translates commands from the *DCS* communication language to the individual languages of the various peripheral control systems. DHSs can be written in any computer programming language and act as thin clients to translate commands between DCS and myriad control subsystems. A typical synchrotron beamline can have upwards of a dozen computers running different operating systems. Each computer is responsible for controlling a subset of the various motors, actuators, ion chambers, robotic hardware, detectors, *etc.* that are required for a functioning beamline/endstation. The control and data acquisition activities performed by the heterogeneous networked computer environment must be tightly coordinated. For example, it is important to open the experimental X-ray shutter before telling the detector to start recording an image. *DCS* communicates simultaneously with all beamline hardware and can be likened to an air traffic controller, using rule-based decision making to coordinate disparate activities from a central ‘clearing house’. The beamline user sees and controls only the hardware pertinent to the experiment *via* the *Blu-Ice* graphical user interface (GUI). Although it is possible for many instances of *Blu-Ice* to be connected to the DCSS (Fig. 1 ▶), only one instance of *Blu-Ice* can be ‘active’. This ensures that only one person at a time is controlling the experiment. Collaborating scientists can have instances of *Blu-Ice* open to monitor the ongoing experiment in real time. At any point control of the beamline can be toggled on/off by clicking on the active/passive button located at the bottom of all *Blu-Ice* windows. Traditionally *Blu-Ice*/*DCS* was developed for MX beamlines, and as such has gradually developed as a full featured but highly specialized macromolecular crystallography tool.

The ability of *Blu-Ice*/*DCS* to function as a control system for SAXS experiments was not immediately feasible. Our goal was to leverage the power and flexibility of the *Blu-Ice* /*DCS* system and adapt it for the high-throughput biological SAXS endstation at the SIBYLS beamline. Although there are fewer motors to deal with and there is no need to coordinate the rotation of the ϕ axis with the opening and closing of the shutter as is the case with an MX endstation, there were nonetheless significant challenges to adapting *DCS* and *Blu-Ice* to a SAXS data collection environment. The primary challenge was to assure the accuracy of the shutter timing and the concurrent acquisition of critical ion-chamber information that is used to normalize and process the data. Our goal was to efficiently integrate control of the liquid-handling robot with the collection of SAXS data. Because *Blu-Ice*/*DCS* was designed to be portable, modular and relatively easily adapted, this task was practicable.

## 2. *Blu-Ice* for SAXS

*Blu-Ice* is the GUI for users to set up, monitor, evaluate and control their experiments. The general features, design and use of *Blu-Ice* for MX have been well documented elsewhere (McPhillips *et al.*, 2002 ▶; Soltis *et al.*, 2008 ▶; McPhillips, 2009 ▶). The *Blu-Ice* GUI is a single graphical window written in Tcl/Tk and as such is easily modified and extended without recompiling between changes. The main *Blu-Ice* window consists of multiple tabs which contain the controls and graphical widgets for a particular set of tasks. Because of the relative simplicity of a SAXS experiment compared with an MX experiment, many of the original tabs available in the MX version of *Blu-Ice* have been removed. For example, the MX Hutch tab provides the user with a view of the most relevant motors of the beamline. These include the detector distance, X-ray energy, beam size, goniometer axis, *etc.* At our SAXS endstation many of these motors do not exist or users do not need to routinely move them. Of the motors accessible *via* the MX Hutch tab of *Blu-Ice* the only motor relevant to SAXS experiments at the SIBYLS beamline is the X-ray photon energy, and this is only to be changed by the beamline scientist, as non-trivial re-optimization of the beam focus is required after the change in energy. However, some SAXS beamlines may have the ability to move detector distance and as such *Blu-Ice* is readily configurable. Additionally there is no need for control and acquisition of fluorescence data so the MX Scan tab has also been removed. As a result we have trimmed the number of *Blu-Ice* tabs to just five (Collect, Screening, Users, Log and Staff). These are described in further detail below.

### 2.1. Collect tab

**Figure 2:**
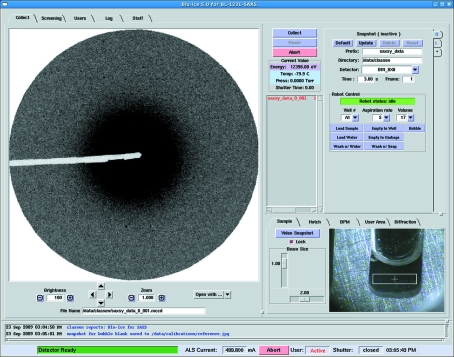
Collect tab of *Blu-Ice* for SAXS. Individual experimental run tabs (0, 1 and *) are located on the right side of the Collect tab. The snapshot tab (Tab 0) allows individual control of the sample-loading robot and the detector. Fully automated experimental runs are defined in run tabs 1–14. New tabs are generated by clicking on the * tab. A video widget allows the user to watch their sample as it is loaded into the cell, and an image-viewing window shows the most recent scattering data in the left side of the window.

The Collect tab is used to set up, control and monitor individual SAXS data collections (Fig. 2 ▶). The SAXS Collect tab has been modeled after the MX Collect tab. The left pane contains a diffraction image viewer that displays a compressed JPG image generated from the raw MarCCD tiff image. There are controls for adjusting the brightness of the image, translating to different regions, and zoom controls. Additionally, if a user double-clicks on the scattering image it will be opened in *ADXV* (Area Detector Systems Corporation; Poway, CA, USA) (Arvai, 2009 ▶). This allows users to inspect in more detail the original image file. The lower right pane contains a tabbed video widget with four video tabs for viewing the sample cell, the SAXS endstation inside the hutch, the live beam-positioning monitor, and the user area. The video image of the SAXS sample cell is useful for monitoring the progress of the Hamilton sample-loading robot and determining whether the sample contains a bubble or has been loaded satisfactorily for subsequent data collection. The upper right pane contains two sub-panes. The left sub-pane contains the primary control buttons for starting, pausing and aborting the current experiment. It also contains a small window that reports the X-ray photon energy and the current pressure and temperature of the MarCCD detector, and a dynamic list that informs the user of the exposures that are configured for collection within the current tab.

The rightmost sub-pane contains a vertically tabbed notebook with room for up to 16 experiment tabs (Fig. 2 ▶ right-hand side). Tab 0 is a special snapshot tab that allows independent control of both the detector and the Hamilton sample-loading robot, whereas tabs 1–16 are regular data collection tabs that can be configured to automatically control both the sample-handling robot and the detector. In the lower portion of tab 0 there is a Robot Control widget with pulldown menus for selecting well number (A1–H12), aspiration rate (0–20 µl s^−1^) and volume (1–20 µl). These values are then used by the control buttons for commanding the robot to perform one of seven pre-programmed actions. These include Load Sample, Load Water, Wash with Water, Empty to Well, Empty to Garbage, Wash with Soap, and Bubble. These methods are self-explanatory with perhaps the exception of the ‘Bubble’ command. Although bubbles occur rarely, they can make measurements from a sample useless. Therefore, the Bubble command was designed as a way to dislodge bubbles introduced to the sample cell during the Load Sample operation.

During a Bubble operation the Hamilton robot inserts the sample-loading needle (1 mm diameter) to the bottom of the sample cell (2 mm thick) and moves back and forth four times. If there are a few large bubbles this mechanical action dislodges bubbles and they float to the top of the sample and out of the X-ray beam path. Multiple bubbles or single small bubbles are not always successfully removed. We have developed a machine-vision-based method for detecting the presence of bubbles (see §3.4 below) which flags bubble-containing samples for subsequent manual inspection and intervention.

Tabs 1–16 are ‘Run tabs’ which allow automatic sample loading and data collection for up to 16 successive samples. A run is a sequence of exposures taken on a single sample. For each run a user may specify the prefix of the image file names, the directory where the data should be saved, and the binning mode of the detector. Up to five exposure times may be requested per sample. Again pulldown menus are used to select well number, aspiration rate and volume. For each run the user may select up to eight steps to be performed by the robot. For each step there is a pulldown menu offering the seven preprogrammed robot methods plus one additional method called ‘Expose’. The exposure times (up to five) are collected in rapid succession during the Expose step. An example run might be set up as follows: (1) Load Sample, (2) Bubble, (3) Expose, (4) Empty to Well, (5) Water Wash. Before each Expose step a video snapshot is taken of the sample well so that users may inspect the sample at a later time.

### 2.2. Screening tab

**Figure 3:**
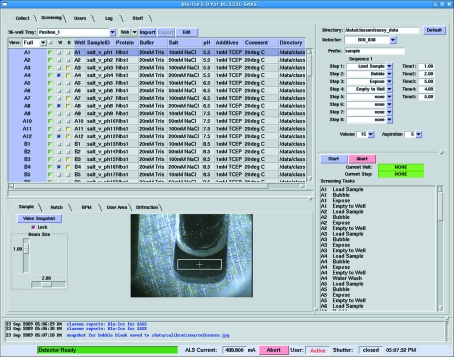
Screening tab of *Blu-Ice* for SAXS. The spreadsheet defining the SAXS experiment is imported into the upper left panel, screening actions to be performed on each sample are defined in the upper right, scheduled tasks are dynamically updated in the lower right, and the video widget is located in the lower left panel.

**Figure 4:**
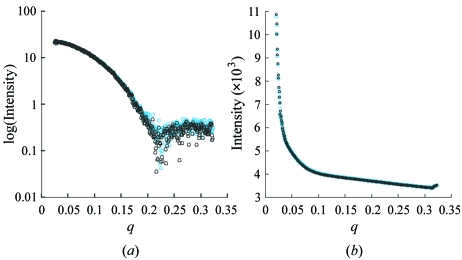
Control demonstrating efficient removal of solution by liquid-handling robot. (*a*) Data collected from a 3 mg ml^−1^ xylanase solution with a matching buffer collected before and after the protein solution. No washing steps were performed between exposures. The blue curve has the first buffer subtracted and the black curve has the second buffer subtracted. (*b*) Raw data from the two buffers have been overlaid to show that there is no carry over of the xylanase when measuring the second buffer.

The Screening tab is used to set up, control and monitor high-throughput SAXS data collection experiments (Fig. 3 ▶). The left pane contains a spreadsheet listing all 96 wells of the sample plate. A check box next to each well label allows any combination of the 96 samples to be selected for screening. Additionally, check boxes next to each sample indicate that the sample is a buffer or specify whether the sample cell should be washed after data are collected. Users obtain visual confirmation that the robot is functioning and that the sample contains no bubbles by way of the video widget located directly below the sample selection list. The upper right pane contains the parameters that define the actions to be performed for each sample selected in the spreadsheet sample selection list. The ‘Wash’ step is the bottleneck in data collection (taking ∼3 min to complete) and, since the user may be collecting data from a series of diluted samples (lowest concentration to highest concentration), it may not be necessary to wash after each sample (Fig. 4 ▶). Therefore we have added a check box so users can optionally select which steps should not be followed by a wash cycle. Although this greatly speeds up the SAXS experiment by avoiding potentially unnecessary wash steps, for the absolute highest confidence in the data it is recommended that users wash the sample cell between each and every buffer and sample. As with the Collect tab the user defines the directory where data should be saved, the desired binning mode of the detector, and the prefix to use for naming the files. The user then programs the commands (up to eight) that will be carried out for each sample. A summary of programmed steps is dynamically updated in the screening tasks pane located directly below the screening actions pane. This gives instant feedback to the user as they design their data collection and allows for visual confirmation that the run is being configured as expected.

### 2.3. Users tab

The Users tab indicates who is currently logged into the SAXS endstation and how many instances of *Blu-Ice* are currently open. *Blu-Ice* has been designed so that only one instance of *Blu-Ice* can control the beamline at any one time. The Users tab indicates the active user by changing the text to red and placing a small arrow next to the users name. This is particularly useful for remote data collection experiments where multiple users from around the world might be logged into the beamline at the same time while collaborating on a project. Additionally, there may be a beamline scientist at home who is supporting the user. The Users tab allows everyone to see who is currently commanding the beamline.

### 2.4. Log tab

The Log tab keeps a running record of the date, time, data collection and robot steps, and file names of all data collected during a users shift. Additionally, the log file contains the results from the bubble detection algorithm (§3.4 below). This is an important consideration when processing SAXS data and having these results readily available in the Log tab allows users to quickly ascertain whether data will be negatively affected by bubbles. Additionally, the data file containing the associated X-ray intensity information for each image is listed here. The users log file can be easily saved into the users home directory and can be used to guide subsequent SAXS data processing steps.

## 3. SAXS specific modifications

In order to better accommodate different beamline requirements and configurations, *DCS* provides scripted operations which allow efficient implementation of arbitrarily complicated beamline operations using custom Tcl scripts (McPhillips *et al.*, 2002 ▶). Additionally, by utilizing the *DCS* communication protocol, *Blu-Ice*/*DCS* beamline controls can be extended to include any hardware component through the incorporation of additional DHS modules, as long as the hardware provides a mechanism of communication (TCP/IP, RS232, *etc.*). We have written a number of scripted operations and DHSs to make *Blu-Ice*/*DCS* a full featured SAXS beamline control system. Some of these efforts are detailed below.

### 3.1. MarCCD detector DHS

The detector DHS is specialized C/C++ code that communicates between DCSS and the MarCCD 165 detector installed at the SIBYLS SAXS endstation. The detector DHS code was provided by SSRL and was modified to allow the collection of 2×2, 4×4 or 8×8 hardware binned images. Solution X-ray scattering features are distributed over large solid angles relative to pin point diffraction peaks and powder rings. Thus for solution scattering, higher binning modes (8×8) reduce the contribution of CCD read-out noise, as the binning is done before digitization (*i.e.* the noise of one readout event is spread over an area of 8×8 pixels instead of each pixel getting its own readout noise). However, when analyzing certain data from a SAXS experiment in which there are suspected to be features smaller then 1 mm as measured on the face of the detector, such as rings from powder or crystallites in solution, it is desirable to have the option of collecting 2×2 or 4×4 binned data. Also, when investigating super low-angle features near the beam stop, 2×2 or 4×4 frames are preferable. The modifications we have made to the detector DHS code allow the user to easily switch between binning modes.

### 3.2. Expose operation

**Figure 5:**
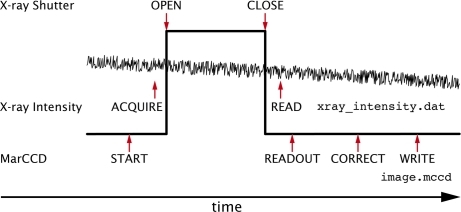
Schematic timeline depicting how the expose scripted operation coordinates the X-ray shutter, detector and the acquisition of critical X-ray intensity data.

During SAXS data collection it is critical that the integrated dose of each image and its corresponding blank are precisely known for the time that the X-ray shutter is open (Fig. 5 ▶). Because the same sample cell is used for collecting the sample and blank data (and we only have one X-ray beam and one detector), they cannot be measured simultaneously. Even at relatively stable synchrotron X-ray sources changes in the intensity of the X-ray beam can easily be larger than 10^−3^ or 10^−4^ during the time in which samples are measured. The resulting scattering, therefore, must be carefully normalized to the intensity of the incident X-rays. For this purpose we have written a scripted operation which starts the acquisition of critical X-ray intensity signals from *I*_mono_ (located just after the monochromator), *I*_zero_ (located in the helium box immediately upbeam from the sample chamber) and *I*_end_ (measured by a PIN diode built into the beam stop). These data are recorded every 0.5 ms (2000 Hz) and written to a file concurrent with the sample exposure (Fig. 5 ▶).

### 3.3. Screening operation

The Screening tab represents the culmination of the SAXS beamline controls, and although this functionality has been implemented in the MX Screening tab provided by SSRL we have extensively re-written this operation to support automated SAXS data collection. The scripted operation that is called from the Screening tab controls the automatic collection of data from up to 96 individual samples and buffers. This scripted operation sequentially loads and unloads samples, collects matched sample and background images, washes the well between samples, and checks for the existence of bubbles. By the time the experimenter has programmed the run and pressed the ‘Collect’ button the entire set of commands has been stored in the DCSS memory. The operation of all screening tasks is now essentially under control of DCSS. The *Blu-Ice* GUI may then be closed (purposely or accidentally) and the screening operation will continue. Additionally, within DCSS there are internal checks for the presence of stored beam, whether the experimental shutter is opening and closing as commanded, or whether the robot fails to complete an operation. For simple errors like the beam dumping, DCSS will pause the data collection until light is restored, at which time data collection will automatically resume. In the presence of serious errors, DCSS will send an email notification and text message to the beamline scientist in charge, and pause data collection.

Timing measurements for the screening operation are highly dependent on the details of the commanded steps such as length and number of exposures and how often washing steps are performed. However, we have measured SAXS data at the SIBYLS beamline from 96 wells in ∼3 h using a typical screening procedure of Load Sample, Bubble, Expose (0.5 s, 5 s, 0.5 s), Empty to Well, followed by a Wash step after every fourth well. This procedure assumes you are collecting from buffer followed by three identical samples having successively increasing concentrations followed by a wash step before the next buffer/sample set is measured. Additionally, the monochromator at the SIBYLS beamline is equipped with multilayer optics which provide ∼40× more flux than conventional Si(111) optics, and allow for very short exposure times, so this ‘typical’ screening procedure is specific to our beamline.

It can be time-consuming to manually program the Screening tab from within *Blu-Ice*, therefore we have written an import/export utility which allows users to design SAXS screening experiments in any readily available spreadsheet program, where the use of cut-and-paste among other spreadsheet tools makes quick work of experiment design and layout. The resulting *.xls file can then be imported into the Screening tab of *Blu-Ice*. The user can take notes and modify the spreadsheet within *Blu-Ice* and, when finished with the SAXS experiment, can export the results for further analysis and for re-use during subsequent SAXS data collections. Because of the high-throughput nature of the SAXS screening experiments it is important to keep meticulous notes about the solution conditions for each sample. SAXS data and their subsequent analysis are intricately linked with the buffer conditions of the sample. By formalizing the experimental design and data collection in spreadsheets, data are permanently linked to the notes regarding sample preparation as well as buffer conditions and other relevant experimental parameters. Ultimately this information can more easily be added to SAXS structural databases such as BIOISIS (Rambo, 2009 ▶).

### 3.4. Bubble detection

**Figure 6:**
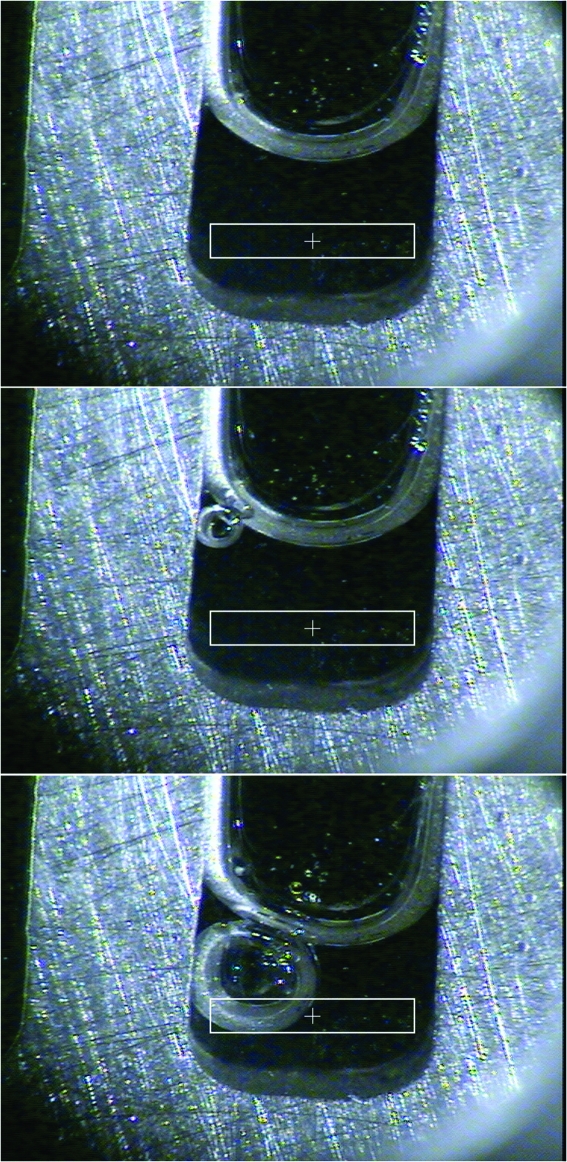
Bubble detection. The top image is a bubble-free sample. The middle image shows an example of a bubble that is outside of the ROI and will not affect the SAXS experiment (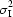 ≃ 400). The bottom image shows an example of a bubble that would be very problematic for collecting high-quality SAXS data (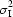 ≃ 2000).

**Figure 7:**
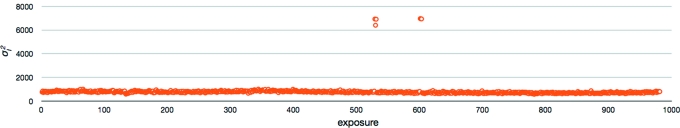
Bubble factor. This graph illustrates the stability of the bubble factor (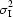) over a 24 h period during which time 980 exposures were collected. During the collection there was one sample, beginning at exposure #533, that contained multiple bubbles in the ROI and a second sample, beginning at exposure #602, that was incompletely loaded with the meniscus located within the ROI.

Obtaining the best possible SAXS data requires that no bubbles are present in the region of the sample exposed to X-rays. From our experience with many users we have found that bubbles occur despite meticulous care taken by the experimenters. The occurrence of bubbles is partly sample dependent and when they occur it is important to identify them and alert the user. We have implemented a warning system to alert users of the possible presence of bubbles. The beamline scientist defines the region of the sample cell corresponding to the size and shape of the X-ray beam [the region of interest (ROI)] by clicking the mouse in the video tab to center a rectangular box. The size of the ROI is adjusted with slider controls located to the left of the video image. During automated data collection an image of the sample cell is taken before each exposure (Fig. 6 ▶). The image is cropped to the ROI, converted to greyscale, and a variance of intensity (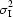) is calculated using the histogram functionality operation from the Kansas University Image Processing System (KUIM) (Gauch, 2009 ▶). We have determined empirically that 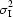 ≥ 2000 indicates a high probability that a bubble is present, a value between 1500 and 2000 indicates there is likely to be a bubble or some other piece of dust and the images should be inspected before processing the data, and a value of ∼400 can safely be assumed to have no bubbles present (see Fig. 7 ▶ for an example). In the current implementation of the bubble-detecting algorithm, samples containing bubbles are flagged in the user’s log file. Because typical data collection times are very short (0.5 s to 10 s), aborting the data collection because of the suspected presence of a bubble will not save much time.

## 4. Remote SAXS data collection

The combination of the Hamilton liquid-handling robot capable of rapidly loading samples from a standard 96-well plate and *Blu-Ice* for SAXS has made it possible for remote users to collect SAXS data or to actively monitor and collaborate with users at the beamline. The SIBYLS beamline has installed an NX Server (freenx) on the main SAXS data collection computer. Remote users can connect using the freely available NX client. NX clients are available for all major operating systems (Linux/UNIX, OS X and Windows). Once connected, the remote user is presented with the same Linux desktop they would see if actually sitting at the beamline. The NX server/client is optimized to handle remote X11 sessions and through efficient compression algorithms provides a very responsive remote computing and data collection experience. As previously mentioned, the *Blu-Ice*/*DCS* system is designed to only allow one instance of *Blu-Ice* to have control of the beamline. Control is handed off from one *Blu-Ice* to another by users toggling the ‘active/passive’ button located at the bottom of each *Blu-Ice* window.

## 5. Conclusions and future directions

We have presented our implementation of software automation and integration tools for the collection of high-throughput SAXS data using the *Blu-Ice*/*DCS* control system which has made the collection of SAXS data easier, less error prone, more efficient and more reproducible. The *Blu-Ice*/*DCS* system was first made available to users in late 2008. In the first full year for which we have statistics (2009) there were 200 eight-hour SAXS shifts during which time 69 external users successfully used *Blu-Ice* to collect their data. For new users the initial training takes about an hour, at the completion of which they can collect data unsupervised. Although we are able to provide remote automated data collection, and have done so on several occasions, we are currently operating almost entirely in an attended mode whereby beamline scientists or users are physically present while collecting data. Our future goals include increasing the capacity of the sample-loading robot and beginning to integrate data analysis tools so that users receive instant feedback about the quality of the data. Overall we expect that providing software that is robust and also familiar to crystallographic users will encourage joint SAXS and MX experiments. Such hybrid methods are increasingly able to solve critical biological problems, as shown for example by recent results on drought receptor ligand binding (Nishimura *et al.*, 2009 ▶), DNA double-strand break repair (Williams *et al.*, 2008 ▶, 2009 ▶; Shin *et al.*, 2003 ▶), DNA base repair (Bernstein *et al.*, 2009 ▶), map kinase regulation (Min *et al.*, 2009 ▶) and reactive oxygen defense enzyme assembly (Shin *et al.*, 2009 ▶).
